# Effects of *Lactobacillus rhamnosus *GG supplementation on cow's milk allergy in a mouse model

**DOI:** 10.1186/1710-1492-7-20

**Published:** 2011-12-06

**Authors:** Cin L Thang, Bushansingh Baurhoo, Joyce I Boye, Benjamin K Simpson, Xin Zhao

**Affiliations:** 1 Department of Animal Science, Macdonald Campus, McGill University, 21,111 Lakeshore, Ste Anne de Bellevue, Quebec, H9X 3V9, Canada; 2 Food Research and Development Centre, Agriculture and Agri-Food Canada, 3600 Casavant Blvd. West, St-Hyacinthe, Quebec J2S 8E3, Canada; 3 Department of Food Science and Agricultural Chemistry, Macdonald Campus, McGill University, 21,111 Lakeshore, Ste Anne de Bellevue, Quebec, H9X 3V9, Canada

## Abstract

**Background:**

Cow's milk allergy (CMA) is one of the most prevalent human food-borne allergies, particularly in infants and young children from developed countries. Our study aims to evaluate the effects of *Lactobacillus rhamnosus *GG (LGG) administration on CMA development using whole cow's milk proteins (CMP) sensitized Balb/C mice by two different sensitization methods.

**Methods:**

LGG supplemented mice were either sensitized orally with CMP and cholera toxin B-subunit (CTB) as adjuvant, or intraperitoneally (IP) with CMP but without the adjuvant. Mice were then orally challenged with CMP and allergic responses were accessed by monitoring hypersensitivity scores, measuring the levels of CMP-specific immunoglobulins (IgG1, IgG2a and IgG) and total IgE from sera, and cytokines (IL-4 and IFN-γ) from spleen lysates.

**Results:**

Sensitization to CMP was successful only in IP sensitized mice, but not in orally sensitized mice with CMP and CTB. Interestingly, LGG supplementation appeared to have reduced cow's milk allergy (CMA) in the IP group of mice, as indicated by lowered allergic responses.

**Conclusions:**

Adjuvant-free IP sensitization with CMP was successful in inducing CMA in the Balb/C mice model. LGG supplementation favourably modulated immune reactions by shifting Th2-dominated trends toward Th1-dominated responses in CMP sensitized mice. Our results also suggest that oral sensitization by the co-administration of CMP and CTB, as adjuvant, might not be appropriate to induce CMA in mice.

## Background

Cow's milk allergy (CMA), an immunologically mediated reaction to cow's milk proteins [1], is one of the most prevalent human food-borne allergies, particularly in infants and young children. In North America, incidence of CMA is estimated at 2.5% in children and about 1% in adults with a 75% outgrowing rate at 16 years of age [2]. Milk protein comprises a mixture of multiple proteins, including whey (such as β-lactoglobulin, α-lactalbumin and bovine serum albumin) and casein (such as α-S1-, α-S2-, β-, κ-, and γ-caseins) proteins. Hypersensitivity reactions may occur upon exposure to a single or multiple milk protein(s). Numerous attempts have been made to reduce or eliminate the allergenicity of milk proteins. Of these attempts, most have focussed on two approaches: to alter the structure and property of milk proteins through thermal treatments, biochemical processes (enzymatic digestion), irradiation [3] and high pressure treatments [4], and to modulate immune responses through sensitization and tolerance induction by means of controlled exposure to a specific allergen which is commonly referred to as specific immunotherapy [5]. Nevertheless, total avoidance of cow's milk or its associated products still remains as the best remedy for CMA. Hypersensitivity to orally ingested food usually occurs upon failure to induce oral tolerance. Research with germ-free mice has indicated that the interaction between allergens and host's gut microbiota plays a crucial role in oral tolerance development [6] and in reducing secretions of allergen-specific antibodies [7]. The gut microbiota is also reported to favour anti-allergenic reactions by mediating T-helper-1 (Th1) type of immunity [8] or inducing IL-10 and transforming growth factor-β (TGF-β) that suppresses T-helper-2 (Th2) type of immunity [9]. Recently, delayed microbial exposure and/or reduced diversity of the gut microbiota among children have been associated with higher allergy incidences [10]. This concept was first reported by Strachan [11] and later widely known as the 'hygiene hypothesis'. Interestingly, whereas the gut microbiota of allergic infants contained higher levels of *Clostridia*, intestinal *Lactobacilli *and *Bifidobacteria *were more predominant among healthy infants [12,13]. Such findings have triggered considerable scientific interests in probiotics, particularly *Lactobacilli *and *Bifidobacteria*, for prevention or treatment of allergies among infants. The allergy reducing effects of probiotics against food allergens such as egg ovalbumin [14,15] and whey proteins [16] have been demonstrated in mouse allergy models. But, to the best of our knowledge, probiotic effects of *Lactobacillus rhamnosus *GG (LGG) to reduce or control allergy to whole cow's milk protein (CMP) have not yet been reported in a mouse allergy model. We used the Balb/C mice model based on its similarity with the human immune system, particularly the Th1 and Th2 responses [17].

Oral sensitization is well recognized as an ideal route to investigate allergic responses to food allergens. Because mice usually develop oral tolerance and fail to manifest allergic responses to ingested allergens, allergens are frequently co-administered with an adjuvant. However, recent reports indicate that commonly used adjuvants, such as cholera toxin (CT) and alum, possess immune-stimulatory properties that may falsely test non-allergenic food products as positive [18]. Consequently, there is increasing interest to develop adjuvant-free systemic sensitization models for testing food allergenicity in mice. The main objectives of this study were to evaluate probiotic effects of LGG on CMA development in a Balb/C mouse model using either an adjuvant-assisted oral sensitization (CMP with cholera toxin B-subunit, CTB) method or an adjuvant-free systemic sensitization (CMP only) method.

## Materials and methods

### Cow's Milk Proteins

Cow's milk proteins were prepared from fresh milk. Briefly, milk was defatted by centrifuging at 1,000 g for 10 min at 4°C and discarding the upper fat layer [19]. After addition of 12% trichloroacetic acid (TCA) (w⁄v), milk proteins were allowed to precipitate for 2 h at 4°C before centrifuging at 9,300 g for 10 min at 4°C. The supernatant was discarded and equal volume of distilled water was added more than five times to the precipitated whole milk protein to remove excess TCA. The concentrated CMP was then lyophilized and stored at 4°C. CMP's protein content (82.34 ± 0.53%) was verified by the Kjeldahl method whereas the presence of major milk proteins was confirmed by 12% SDS-PAGE gel electrophoresis.

### Mice

Three weeks-old female Balb/C mice were purchased from Charles River Breeding laboratories (St. Constant, Quebec, Canada). All mice were fed a diet that was free from animal proteins and microbes (Harlan Teklad, Madison, WI, USA). Feed and water were provided ad libitum. Mice were raised in individual cages, and under a 12L:12D lighting cycle, 20-24°C range of ambient temperature and 40-70% of relative humidity. The animal use protocol was approved by the McGill University Animal Care Committee.

### Sensitization and Challenge Procedures

#### Intragastrically Sensitized (Gavage) Group

**Figure 1:**
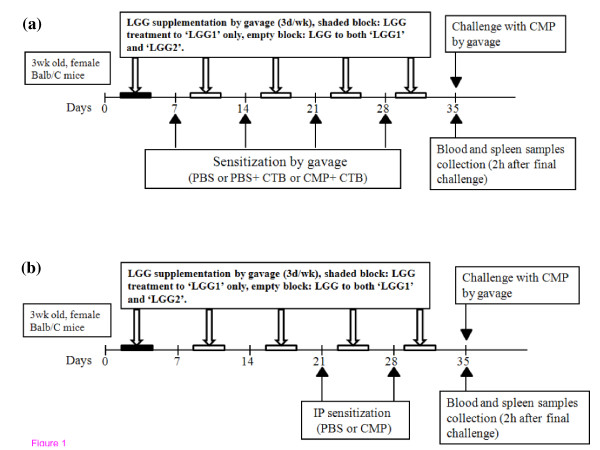
**Schematic overview of CMP sensitization and challenge protocol in Balb/C mice**. **(a) Intragastrically sensitized (gavage) group**. Mice were sub-divided into 5 treatments and sensitized intragastrically for 4 weeks as follows (n = 6); **CTL-: **PBS; **CTB: **PBS+ CTB (0.25 μg/g BW); **CTL+: **CMP (1 mg/g BW) + CTB; **LGG1: **CTL+ mice supplemented with LGG for 5 weeks and **LGG2: **CTL+ mice supplemented with LGG for 4 weeks. At d 35, all sensitized mice were intragastrically challenged two times with CMP (30 mg/mouse) at 30 min apart. Two hours after final challenge, mice were euthanized with CO2 inhalation and blood and spleen samples were collected. **(b) Intraperitoneally sensitized (IP) groups**. Mice were sub-divided into 4 treatments and sensitized intraperitoneally for 2 weeks as followed (n = 6); **CTL-: **PBS; **CTL+: **CMP (10 mg/mouse); **LGG1: **CTL+ mice supplemented with LGG for 5 weeks and **LGG2: **CTL+ mice supplemented with LGG for 4 weeks. At d 35, all sensitized mice were intragastrically challenged two times with CMP (30 mg/mouse) at 30 min apart. Two hours after final challenge, mice were euthanized with CO2 inhalation and blood and spleen samples were collected.

The experimental design for the CMP intragastrically sensitized group is shown in Figure 1a. At 4 weeks of age, mice were sub-divided into 5 experimental sub-groups (n = 6/group) which included mice gavaged with: PBS (CTL-), PBS + CTB [0.25 μg/g BW] (CTB), and CMP [1 mg/g BW] + CTB (CTL+) in a total volume of 200 μl at weekly intervals over 4 consecutive weeks. The last 2 treatments, namely LGG1 and LGG2, included mice from the CTL+ sub-group that were orally treated with a viable dose of LGG (1 × 10^9 ^CFU/day) over 3 days per week. Mice in the LGG1 sub-group received their first LGG dose at 23 d which lasted over 5 weeks whereas LGG2 received LGG from 31 d of age over 4 weeks. The LGG dosage was adopted from a dose-response study with LGG strain HN001 in mice [20]. Four weeks after the first sensitization, mice in all treatment groups were orally challenged with CMP as previously described [19] with minor modifications. Briefly, mice were fasted overnight and challenged with two doses of CMP (30 mg/mouse) at 30 min interval. Two hours after the last dose, mice were euthanized by carbon dioxide asphyxiation. Blood was collected by intracardiac puncture and then spleens were aseptically excised and cryopreserved for later cytokine analyses.

#### Intraperitoneally Sensitized (IP) Group

The experimental design for CMP intraperitoneally sensitized group is shown in Figure 1b. There were 4 treatments in the IP group of mice which included CTL-, CTL+, LGG1, and LGG2 similar to the gavage group of mice. At 6 and 7 weeks of age, each mouse in the 4 treatment groups received 2 doses of CMP (10 mg/mouse) dissolved in 250 μl of PBS intraperitoneally. One week after the second IP sensitization, mice were orally challenged with CMP, and blood and spleen samples were collected similar to mice of the gavage group.

### Evaluation of Hypersensitivity Symptoms

Within an hour following the final oral CMP challenge, hypersensitivity symptoms were scored by a person blind to the study using the score system as described by Schouten et al. [21]. The scores were as follows: 0 = no symptom; 1 = scratching and rubbing around the nose and head; 2 = reduced activity; 3 = activity after prodding and puffiness around the eyes and mouth; 4 = no activity after prodding, laboured respiration, and cyanosis around the mouth and the tail; and 5 = death.

### Determination of Serum CMP-specific (IgG1, IgG2a and IgG) and Total IgE

Blood samples from mice in the gavage and IP groups were collected into serum separator tubes (Sarstedt, Montreal, Quebec, Canada). The serum portion was separated by centrifugation at 10,000 × g for 5 min at 20°C. Serum samples were then aliquoted into eppendorf tubes and stored at -20°C until being analysed. CMP-specific serum immunoglobulins, namely IgG1, IgG2a and IgG as well as total IgE, were detected by ELISA. Briefly, 96-well plates (Dynatech Laboratories, Chantilly, VA) were coated with 100 μg/mL of CMP in 0.1 mol/L Na-bicarbonate/carbonate coating buffer (pH 9.6). After overnight incubation at 4°C, plates were washed 3 times with 150 μl of PBS plus 0.05% Tween-20 (PBS-T) and blocked with 100 μl of 2% fish gelatine (Sigma Aldrich, Ontario, Canada) in PBS-T for 1 h at 37°C. Subsequently, the plates were washed 3 times and 100 μl of serially diluted serum samples (started from1:10 dilutions for IgE, IgG1 and IgG2a and 1:100 for IgG) were added to the wells and incubated at 37°C for 90 min. Plates were then washed 3 times, and 100 μL of horseradish peroxidase (HRP) conjugated anti-mouse antibodies (1:2000 for IgE, IgG1 and IgG2a and 1: 4000 for IgG) were added to each well. The plates were again incubated at 37°C for another 60 min and washed 3 times. Then, 100 μl of ABTS were added to each well and 15 min were allowed for the development of colorimetric reactions. Absorbance was read at a wavelength of 405 nm in a microplate reader (Bio-Tek, Winooski, VT). All analyses were performed in duplicates and the average values were used in the statistical analysis. Serum titres were calculated by the intersection of least square regression of A405 versus logarithm of dilution [22].

### Cytokine Measurements from Spleen Lysates

Spleen lysates were prepared as previously described [23]. Briefly, individual spleens were placed in eppendorf tubes containing 0.5 ml of lysate buffer. The spleen cells were lysed and homogenized by sonication for 30 s on ice. Supernatants were collected after centrifugation at 17,500 g for 10 min at 4°C and stored at -20°C. Interleukin-4 (IL-4) and interferon-gamma (IFN- γ) from spleen lysates were analyzed by commercially available ELISA kits following the manufacturer's protocol (R&D systems, Minneapolis, MN). Total protein content in spleen lysates was determined using the detergent compatible protein assay (Bio-Rad Laboratories Inc., Hercules, CA) and bovine serum albumin as standard. Cytokine concentrations from spleen lysates were expressed as pg/mg of total protein of spleen.

### PCR Identification of Fecal Lactobacillus

For PCR analysis, 100 mg of fecal samples were aseptically weighed, placed in sterile tubes and homogenized in 1.4 mL stool lysis buffer (QIAamp DNA stool kit; Qiagen, ON, Canada). Genomic DNA extraction was performed according to the manufacturer's protocol. DNA was then amplified using the Crimson taq DNA Polymerase with Mg-free Buffer, 25 mM MgCl_2_, 10 mM dNTP and Crimson Taq DNA Polymerase (New England BioLabs, ON, Canada) and *Lactobacillus*-specific primers. The primer-set sequences were as described previously [24]. PCR reactions were performed at 95°C for 2 min and 95°C for 30 s, followed by 30 cycles of 45 s at 60°C and 68°C for 5 min in an Eppendorf Mastercycler EP Gradient 5341 (Fisher Scientific, ON, Canada). Finally, the presence of lactobacillus strains was detected by performing agarose gel electrophoresis 1% (w/v) with the PCR products and using genomic DNA from pure *L. rhamnosus *GG (ATCC53103) as control. The PCR amplicons were visualized under UV light (260 nm) followed by a subsequent SafeView nucleic acid staining (0.5 μg/ml; NBS Biologicals, UK).

### Enumeration of Fecal Lactobacilli

*Lactobacilli *counts were determined from fecal samples of IP mice at 16 and 30 d. At 12 h prior to sample collection, mice were transferred to new cages which were lined with moist paper towels instead of the standard rodent bedding. After fecal sample collection into sterile microbiology bags, mice were returned into their respective treatment cages. Sample homogenization, serial dilutions and culture on bacteria-specific agars were as previously described [25]. Briefly, fresh fecal pellets were diluted 10-folds by weight in buffered peptone water, homogenized, and serially diluted in 0.85% sterile saline solution. *Lactobacilli *were anaerobically cultured on *Lactobacilli *MRS agar for 24 h at 37°C. Bacterial colonies were counted at the end of incubation period. Microbiological analyses were performed in duplicates and the mean values were used in statistical analyses.

### Statistical Analysis

Data were analyzed by a one-way ANOVA using the GLM procedure of SAS (SAS Institute, 2003) except for the hypersensitivity scores data which were analyzed using the Kruskal-Wallis test and SigmaStat software (Systat Software Inc., San Jose, CA). Differences among treatment means were tested using the Scheffe's multiple comparison test. *P*-values ≤ 0.05 were considered significantly different. Results were presented as mean ± standard deviation. All microbiological concentrations were subjected to base-10 logarithm transformation before analyses.

## Results

### Hypersensitivity Responses

**Figure 2:**
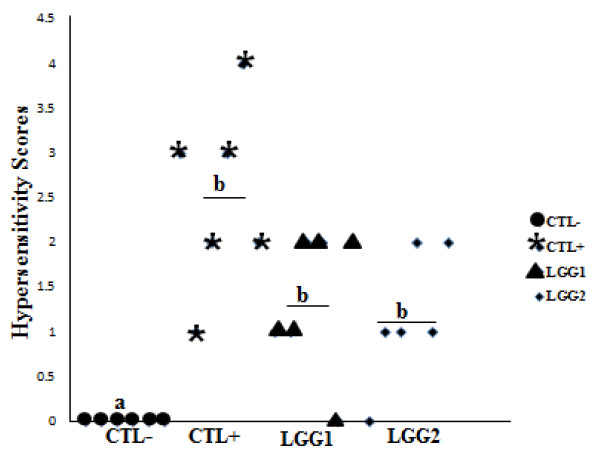
**Hypersensitivity scores of intraperitoneally sensitized (IP) mice**. Hypersensitivity symptoms were scored one hour after last challenge with CMP. Each point represents an individual mouse. Values are means, n = 6 per treatment. **CTL-: **PBS, control mice; **CTL+: **CMP sensitized mice; **LGG1: **CTL+ mice supplemented with LGG for 5 weeks and **LGG2: **CTL+ mice supplemented with LGG for 4 weeks. Means with different letters differ, P ≤ 0.05.

Hypersensitivity scores were recorded within an hour after the final challenge with CMP. In the IP group of mice, a moderate level of discomfort was observed among CMP-sensitized mice in the CTL+, LGG1 and LGG2 treatment groups, while CTL- mice did not show any visible signs (Figure 2). The average scores for hypersensitivity symptoms were 2.5 ± 1.05 in CTL+, 1.33 ± 0.82 in LGG1, and 1.167 ± 0.75 in LGG2, respectively. There were no significant differences in hypersensitivity scores among mice in the CTL+, LGG1 and LGG2 treatment groups. But, in comparison with CTL+ mice, hypersensitivity scores were numerically lowered in LGG1 and LGG2 mice. In the gavage group, however, mice did not show any noticeable hypersensitivity responses (data not shown).

### CMP-specific Immunoglobulin Levels in Serum

**Figure 3:**
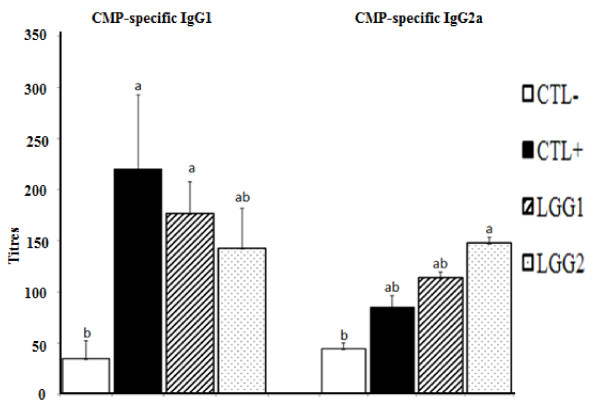
**CMP-specific serum IgG1 and IgG2a titres of IP mice**. **CTL-: **PBS; **CTL+: **CMP (10 mg/mouse); **LGG1: **CTL+ mice supplemented with LGG for 5 weeks and **LGG2: **CTL+ mice supplemented with LGG for 4 weeks. Values are presented as Mean ± standard deviation. Values with different letters in the same column differ, *P *≤ 0.05.

Serum samples were analyzed by ELISA. In the IP group of mice, CMP-specific IgG1, IgG2a and IgG levels were significantly higher in CMP-sensitized (CTL+, LGG1 and LGG2) than non-sensitized (CTL-) mice (Figure 3 and Table 1). Moreover, among CMP-sensitized mice, CMP-specific IgG2a level was higher in LGG2 than CTL+ mice. But, CMP-specific IgG1 levels were lower in both LGG1 and LGG2 in comparison to CTL+ mice. Total IgE levels were similar across all treatment groups (Table 1). But, in the gavage group, CMP-specific IgG1 level did not differ between mice in the CTL+, CTL- and CTB treatments (Table 2).

**Table 1:** Immunoglobulins titres from the sera of IP mice.

Treatments	CMP-specific IgG	Total IgE*
CTL-	71.67 ± 10.75^b^	74.67 ± 28.62
CTL+	101.0 ± 9.07^a^	43.67 ± 14.58
LGG1	102.17 ± 10.64^a^	42.67 ± 5.06
LGG2	112.17 ± 8.76^a^	79.43 ± 48.21

**CTL-: **PBS; **CTL+: **CMP (10 mg/mouse); **LGG1: **CTL+ mice supplemented with LGG for 5 weeks and **LGG2: **CTL+ mice supplemented with LGG for 4 weeks. Values are presented as Mean ± standard deviation. Values with different letters in the same column differ, *P *≤ 0.05.

*Data were not statistically different, P > 0.05.

**Table 2:** CMP-specific Immunoglobulin titres from the sera and cytokines level from spleen lysate in Gavage group.

Treatments	CMP-specific		Cytokine (pg/mg)	
	
	IgG*	IgG1	IL-4*	IFN- γ
CTL-	68.17 ± 4.14	35.33 ± 11.35^b^	9.70 ± 4.64	9.93 ± 0.37^b ^
CTL+	68.0 ± 6.08	34.83 ± 5.87^b^	10.22 ± 5.65	13.65 ± 1.42^ab^
CTB	68.50 ± 3.45	59.50 ± 26.44 ^b^	12.42 ± 6.41	15.52 ± 1.39^ab^
LGG1	76.83 ± 13.98	131.33 ± 51.39^a^	11.90 ± 4.60	14.47 ± 3.20^ab^
LGG2	69.67 ± 5.62	92.17 ± 37.47^ab^	8.42 ± 4.66	16.45 ± 2.26^a^

**CTL-: **PBS; **CTB: **PBS+ CTB (0.25 μg/g BW); **CTL+: **CMP (10 mg/mouse) + CTB; **LGG1: **CTL+ mice supplemented with LGG for 5 weeks and **LGG2: **CTL+ mice supplemented with LGG for 4 weeks. Values with different letters in the same column differ, *P *≤ 0.05. Values are presented as Mean ± standard deviation.

*Data were not statistically different, P > 0.05.

### Cytokine Levels in Spleen Lysates

The IL-4 and IFN-γ concentrations from the spleen of mice in the IP group were not different across treatments (Table 3). In the gavage group of mice, although similar levels of IL-4 were observed in all the treatments, IFN-γ levels were significantly lower in the CTL- mice when compared to mice in the remaining four treatment groups (Table 2).

**Table 3:** Cytokines level from spleen lysate of IP mice (pg/mg of total protein).

Treatments	IL-4 (pg/mg)*	IFN- γ (pg/mg)*
CTL-	15.33 ± 4.51	18.33 ± 3.25
CTL+	19.3 ± 6.36	21.53 ± 5.69
LGG1	17.35 ± 3.56	26.82 ± 8.02
LGG2	14.47 ± 5.07	26.3 ± 4.69

**CTL-: **PBS; **CTL+: **CMP (10 mg/mouse); **LGG1: **CTL+ mice supplemented with LGG for 5 weeks and **LGG2: **CTL+ mice supplemented with LGG for 4 weeks. Values are presented as Mean ± standard deviation.

*Data were not statistically different, *P > 0.05*.

### Fecal Counts and PCR Analysis of Lactobacilli

In the IP group of mice, fecal *Lactobacilli *counts in LGG1 and LGG2 were similar to those in the CTL- and CTL+ groups at d 16 (Table 4). But, at d 30, mice in the LGG2 group had greater *Lactobacilli *counts than CTL- mice. However, *Lactobacilli *counts were similar between CTL+ and LGG1 mice groups.

**Table 4:** Fecal counts of total *Lactobacilli *from IP group at d 16 and d 30.

Treatments	*Lactobacilli*	
	
	d16	d30
CTL-	9.01 ± 0.08	8.70 ± 0.17^b^
CTL+	8.83 ± 0.16	8.89 ± 0.18^ab^
LGG1	9.04 ± 0.12	8.86 ± 0.10^ab^
LGG2	8.80 ± 0.28	9.03 ± 0.13^a^

**CTL-: **PBS; **CTL+: **CMP (10 mg/mouse); **LGG1: **CTL+ mice supplemented with LGG for 5 weeks and **LGG2: **CTL+ mice supplemented with LGG for 4 weeks. Data are presented as the mean log10 colony forming units/g ± standard deviation of fecal sample. For each bacterium, treatment means with different letters in the same column differ, *P *≤ 0.05.

**Figure 4:**
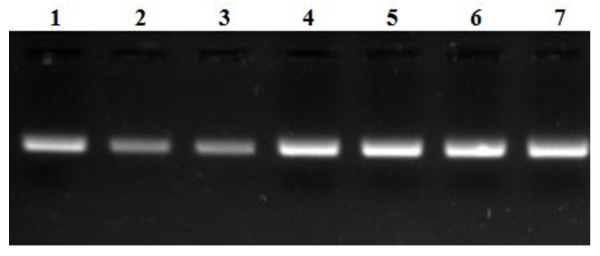
***Lactobacillus rhamnosus *GG (LGG) analysis from fecal genomic DNA using LGG-specific primers **[24]**and subsequent agarose gel electrophoresis**. Lane 1, DNA extracted from supplemented LGG (ATCC53103); lanes 2 and 3, **CTL + mice**; lanes 4 and 5, **LGG1 mice**; lanes 6 and 7, **LGG2 mice**.

PCR analysis was performed on fecal samples to determine whether probiotic-treated mice excreted higher lactobacilli. Indeed, higher concentrations of *lactobacillus*-specific products were detected in fecal samples from LGG-treated mice than mice in the control groups (Figure 4). These findings were indicative of higher levels of the bacteria in the intestinal tract of LGG supplemented mice.

## Discussion

CMA is a global health concern that occurs more frequently among children than adults. In infants, high CMA incidence occurs upon first exposure to CMP, for example through infant formulas, while the immune system is still immature. On the other hand, the intestinal immune-modulating effects of probiotics [26] have been shown to reduce the risks of developing allergic diseases in both mice [14,15,24] and humans [27,28]. The present study evaluated whether oral LGG administration could help reduce or control CMA in Balb/C mice that were sensitized with CMP either via the oral (gavage) or systemic (IP) route. Moreover, to better simulate CMA in infants, we specifically used 3 week-old newly weaned Balb/C mice as an animal model and whole CMP as allergen rather than purified single CMPs.

In the IP group of mice, both LGG1 and LGG2 groups, in comparison with CTL+ mice, may possibly alleviate allergy as indicated by numerically lower hypersensitivity responses (Figure 2), lower IL-4 levels, and lower CMP-specific IgG1 but higher IFN-γ and CMP-specific IgG2a levels (Figure 3 and Table 3). Generally, an increase in Th2 response in mice results in higher secretions of IL-4, and allergen-specific IgE and IgG1, whereas increasing the Th1 response leads to higher IFN-γ and IgG2a levels [29]. Therefore, together with previous in vitro [30] and mice [14,31] studies, our findings suggest that LGG supplementation may alleviate allergic reactions by supressing Th2-mediated immune responses. Similarly, allergy reducing effects by probiotics have been reported in clinical studies [27,28], in which LGG or a mixture of probiotics containing LGG prevented atopic eczema in high risk infants. The anti-allergic effects of probiotics have also been demonstrated in ovalbumin-induced asthma [15] and atopic dermatitis NC/Nga mice models [32]. We also observed that an additional week of oral LGG administration between the LGG1 and LGG2 groups of mice did not significantly alter total *Lactobacilli *counts, hypersensitivity scores, and serum concentrations of IL-4, CMP-specific IgG1 or CMP-specific IgG2a. Therefore, under the conditions of this study, it appears that greater probiotic supplementation beyond 4 time points had no major benefits in alleviating CMA. However, the exact reason is unknown.

Elevated IL-4, allergen-specific IgE and IgG1 are well recognized principal immune mediators of IgE-mediated allergy. Allergen-specific IgE is not easy to be measured when using conventional methods because IgE is generally present at low levels in the serum and its measurement is more complicated by the serum's higher IgG levels. Moreover, it is reported that both CMP-specific IgE and IgG have competitive binding ability to similar epitopes regions of CMP, and that the greater serum IgG levels significantly reduced the binding capacity of serum IgE to CMP [33]. But, in the event that specific IgE is undetectable or non-measurable, IgG1 can be used as a surrogate maker for IgE if it is accompanied by increased IL-4 [34,35]. Although we were unable to detect CMP-specific IgE, our findings about elevated serum IL-4 together with significantly higher CMP-specific IgG1 in CTL+ mice in comparison with CTL- mice indicate that IgE-mediated allergy may have occurred in CMP-sensitized mice. In agreement with our results, CMP-specific IgE was also undetectable in other mice allergy studies [19,36]. It appears that serum concentrations of allergen-specific IgE are highly dependent on the type of administered allergen. For instance, when mice were orally sensitized with casein or whey, Schouten et al. [37] successfully measured whey-specific IgE and IgG1 but could not detect casein-specific IgE. We presume that CMP-specific IgE responses could have been reduced in our study considering the fact that whole CMP contains 20% whey only, but 80% casein. In addition, we suspect that a direct ELISA method used in this study might not have been sensitive enough to detect and measure CMP-specific serum IgE.

Our results about significantly higher hypersensitivity scores and CMP-specific antibodies titres (IgG1, IgG2a and IgG) in CMP-sensitized CTL+ mice with compared to CTL- mice demonstrate that adjuvant-free IP sensitization successfully stimulated CMP-specific immune responses (Figure 2, 3 and Table 1). Therefore, an adjuvant-free systemic sensitization mouse model can well be adopted to study the allergenicity of low allergen-containing foods, considering that adjuvant co-administration may falsely test non-allergic food products as allergic [18].

We did not observe visible hypersensitivity symptoms and different levels of CMP-specific IgG1 and IgG between CTL+ and CTL- subgroups in orally sensitized (Gavage) mice (Table 2). We have chosen CTB due to its non-toxic adjuvant property and it has been used in food allergy studies [38,39]. However, it seems that under the conditions of this study, the oral administration of CMP and CTB mixture was not allergenic to mice. In addition to the well-known fact that oral sensitization could induce tolerance development, recent reports indicate that CTB possesses allergy suppressing rather than stimulating effects in mice through induction of secretory IgA [40]. Based on our findings and the above-mentioned report, it seems that CTB may not be an appropriate oral adjuvant that can successfully induce CMP-specific allergic responses in Balb/C mice.

## Conclusions

To our knowledge, we are the first to investigate the effects of LGG supplementation on CMA in mice that were sensitized with the whole CMP. We believe that the adjuvant-free systemic sensitization model may be particularly useful in the testing of food products with low allergenicity. LGG administration seems to favour suppression of Th2 responses such as reduced hypersensitivity scores and lowered serum CMP-specific IgG1 while promoting Th1 responses by causing elevated IFN-γ and CMP-specific IgG2a levels. Although further experimental and clinical studies are required to elucidate the mechanism involved and complete beneficial effects of LGG, the current study suggests LGG as a potential preventive tool in the fight against CMA.

## Competing interests

The authors declare that they have no competing interests.

## Authors' contributions

CLT, JIB, and XZ designed the research; CLT conducted the research and analyzed the data; BB performed PCR and statistical analysis; CLT and XZ wrote the paper; JIB, BKS and XZ helped to edit the manuscript and CLT and XZ had primary responsibility for final content. All authors read and approved the final manuscript.
